# The Effects of Ethanol on the Morphological and Biochemical Properties of Individual Human Red Blood Cells

**DOI:** 10.1371/journal.pone.0145327

**Published:** 2015-12-21

**Authors:** Sang Yun Lee, Hyun Joo Park, Catherine Best-Popescu, Seongsoo Jang, Yong Keun Park

**Affiliations:** 1 Department of Physics, Korea Advanced Institute of Science and Technology, Daejeon, Republic of Korea; 2 Department of Bioengineering, University of Illinois at Urbana-Champaign, Urbana, Illinois, United States of America; 3 Department of Laboratory Medicine, University of Ulsan, College of Medicine and Asan Medical Center, Seoul, Republic of Korea; Université Claude Bernard Lyon 1, FRANCE

## Abstract

Here, we report the results of a study on the effects of ethanol exposure on human red blood cells (RBCs) using quantitative phase imaging techniques at the level of individual cells. Three-dimensional refractive index tomograms and dynamic membrane fluctuations of RBCs were measured using common-path diffraction optical tomography, from which morphological (volume, surface area, and sphericity); biochemical (hemoglobin (Hb) concentration and Hb content); and biomechanical (membrane fluctuation) parameters were retrieved at various concentrations of ethanol. RBCs exposed to the ethanol concentration of 0.1 and 0.3% v/v exhibited cell sphericities higher than those of normal cells. However, mean surface area and sphericity of RBCs in a lethal alcoholic condition (0.5% v/v) are not statistically different with those of healthy RBCs. Meanwhile, significant decreases of Hb content and concentration in RBC cytoplasm at the lethal condition were observed. Furthermore, dynamic fluctuation of RBC membranes increased significantly upon ethanol treatments, indicating ethanol-induced membrane fluidization.

## Introduction

Ethanol (recreational alcohol) effects on human physiology is an important research topic, given the pervasiveness of alcohol abuse and the myriad of health and social problems associated with heavy drinking. Ethanol and similar anesthetic drugs cause significant alterations of cells, tissues, and organs. In particular, ethanol exposure induces cell membrane remodeling in various cell types and lipid vesicles, including membrane fluidization. Ethanol-induced membrane fluidization, in particular, has been extensively explored using electron paramagnetic resonance (EPR) spectroscopy [1–3] and fluorescence anisotropy [4].

Alcohol-induced modifications in red blood cell (RBC) membranes have been extensively investigated as a model system using diverse experimental techniques that include EPR spectroscopy [1, 5, 6], fluorescence anisotropy [7–11], gas chromatography [12], laser diffraction ektacytometry [6, 13], and micro-size mesh filtration [14]. The ethanol-mediated modifications in hemorheological parameters of RBCs, including mean corpuscular hemoglobin (Hb) concentration (MCHC) and mean corpuscular volume (MCV), were measured with the help of flow cytometry for *in-vitro* ethanol exposures [15] and *in-vivo* ethanol ingestions [15], and from RBCs of chronic alcoholics [9–11]. Ethanol-induced hemolytic behaviors [16–18] and cytoplasmic changes [19] in human RBCs, and accompanied alteration in whole blood fluidity [20, 21] have also been investigated.

However, the previous studies have mainly addressed the ensemble average of hemorheological RBC parameters and thus measurements and analyses of ethanol-induced morphological and biochemical modifications in RBCs were not performed at the individual cell level. In particular, most precedent works on morphological alterations have reported that discernible changes in RBC shape primarily occur at conditions far beyond the physiologically attainable level, e.g. too high blood alcohol concentration or too long incubation time. Their analyses on morphological alterations in RBCs by *in-vitro* ethanol treatments are also qualitative. All these are mainly due to limitations of conventional experimental techniques in quantifying cellular morphologies. Furthermore, preceding studies only addressed morphological, biochemical, or mechanical alterations of the RBCs exposed to ethanol. Accordingly, simultaneous measurements of these alterations, with comprehensive analyses of the correlative relations among them, have not yet been achieved, and hence detailed studies about integrated intracellular mechanisms governing ethanol-RBC interaction are still limited.

Here, we report the results of work in which we performed simultaneous measurement of biochemical, biomechanical, and morphological properties of individual human RBCs in a control group (0.0% v/v) to a lethal (0.5% v/v) ethanol condition. Employing quantitative phase imaging (QPI) techniques [22, 23], interferometric microscopy techniques for measuring optical phase delays, the 3-D refractive index (RI) maps and dynamic membrane fluctuations of RBCs exposed to various ethanol concentrations were systematically measured. From these, we retrieved important RBC parameters including cell volume, membrane surface area, sphericity, Hb concentration, Hb content, and membrane fluctuation, for every measured cell simultaneously.

Our results present that RBCs exposed to moderate ethanol concentrations (0.1 and 0.3%) exhibit lower surface areas and thus higher sphericities than those of normal cells. Significant decreases in Hb content and Hb concentration at the highest ethanol concentration (0.5%)—a dose associated with severe intoxication, and marked by muscular incoordination, blurred vision, stupor, and death—were also observed. In addition, out-of-membrane fluctuations of RBCs at equilibrium states were found to be increased by *in-vitro* ethanol treatments even at lower concentration.

## Results

### Ethanol Effects on RBC Morphology

To obtain 3-D morphologies of individual RBCs at various ethanol concentrations, we employed cDOT (see Materials and Methods). The cDOT provides precise measurements of 3-D RI tomograms and dynamic membrane fluctuations of individual cells [24, 25]. For tomogram measurements, the cDOT records multiple 2-D optical fields of RBCs illuminated at various angles of plane waves, from which 3-D RI maps of the RBCs are reconstructed using the optical diffraction tomography algorithm [26, 27].

**Fig 1:**
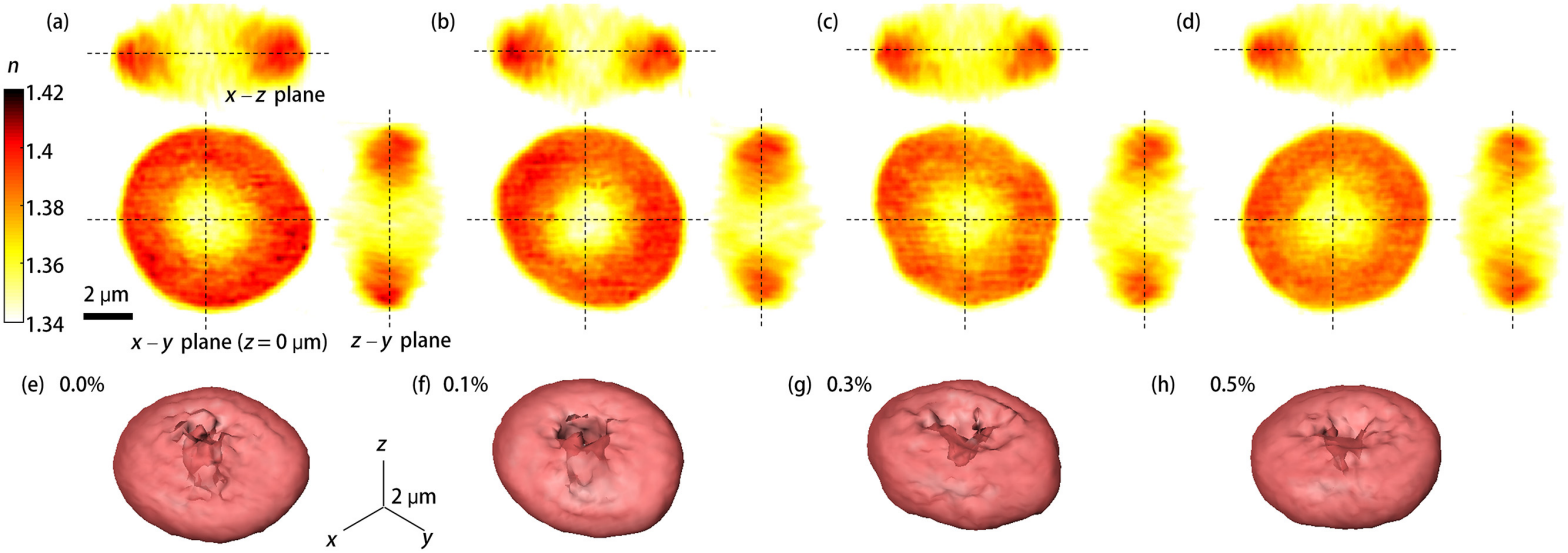
Reconstructed 3-D RI distributions of RBCs. (a–d) Cross-sectional images of 3-D RI tomograms of RBCs (along the *x*–*y*, the *x*–*z*, and the *z*–*y* planes) exposed to diluted blood solutions of (a) 0.0, (b) 0.1, (c) 0.3, and (d) 0.5% ethanol concentrations; (e–h) 3-D rendered RI isosurfaces (*n* > 1.355) of the four representative RBCs in (a–d).

Representative 3-D RI distributions of RBCs exposed to various ethanol concentrations ranging from 0.0 to 0.5% are shown in Fig 1a–1d. Rendered isosurfaces of 3-D RI maps are also shown in Fig 1e–1h for visualization purposes. These reconstructed 3-D RBC structures show that there are no eminent morphological changes in RBCs for the relevant blood alcohol concentration range (0.0 to 0.5%), which is consistent with previous reports [6, 15].

**Fig 2:**
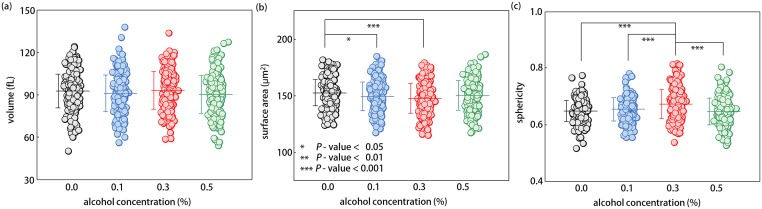
Morphological parameters for RBCs exposed to 0.0, 0.1, 0.3, and 0.5% ethanol concentrations (a) Volume, (b) Surface area, and (c) Sphericity. Each circle indicates individual RBC measurements. Each circle indicates measurements of an individual RBCl measurements. The mean value of each morphological RBC parameters is denoted as the horizontal line with the vertical bar for the standard deviation.

In order to quantitatively analyze the measured 3D RI tomograms, we extracted three morphological parameters including volume, surface area, and sphericity from individual RBCs (Fig 2) (see Materials and Methods).

Our measurements show that there are no statistical differences in mean volume of RBCs belonging to various ethanol groups. Although one-way analysis of variance (ANOVA) rejected the null hypothesis of equal mean volume of RBCs in different ethanol groups with a *p*-value of 0.04, subsequently performed pairwise comparisons with Bonferroni adjustments did not guarantee a lower volume of RBCs in the highest ethanol concentration (0.5% v/v) group than those of the first three ethanol groups (adjusted *p*-value = 0.08 for control group). The mean RBC volumes were 92.7 ± 11.9, 91.0 ± 12.9, 93.0 ± 13.3, and 90.1 ± 13.3 fL for RBCs in the 0.0, 0.1, 0.3, and 0.5% ethanol groups, respectively. Values of the measured cell volumes were within normal physiological range.

Average values of the surface area of RBCs exposed to moderate ethanol concentrations (0.1 and 0.3% v/v) were statistically smaller than those of normal RBCs; yet, mean surface area of RBCs in 0.5% ethanol group had no difference with the normal one. The calculated mean values of surface area were 152.6 ± 11.6, 149.3 ± 12.6, 147.4 ± 13.2, and 150.1 ± 13.1 μm^2^ for RBCs of 0.0, 0.1, 0.3, and 0.5% ethanol concentrations, respectively. Sphericity, a measure of sphere resemblance (see Materials and Methods), directly reflects the observed tendency of the changes in the surface area of RBCs by ethanol. The mean sphericity of RBCs in 0.3% ethanol group was significantly higher than those of other three ethanol groups with *p*-values of less than 0.001. The mean values of sphericity were 0.65 ± 0.04, 0.65 ± 0.04, 0.67 ± 0.05, and 0.65 ± 0.05 for RBCs exposed to the 0.0, 0.1, 0.3, and 0.5% ethanol concentrations, respectively. In both cases, the observed recoveries of both mean surface area and sphericity of RBCs in the 0.5% ethanol group to the normal ranges of healthy RBCs might be not only related to certain abrupt changes in intracellular mechanisms at a lethal alcoholic condition, but also consistent with a previous report about biphasic property of RBC deformability in relevant ethanol concentrations [14]. Values of the measured cell surface area, and sphericity are in good agreement with precedent studies employing micropipette aspiration [28], osmotic feasibility [29], and flow-based imaging [30].

### Ethanol Effects on Biochemical Properties of Individual RBCs

In order to quantitatively and simultaneously investigate the effects of ethanol on RBC cytoplasm, we retrieved the intracellular Hb concentration and Hb content of individual RBCs. Because the Hb concentration is linearly proportional to the RI difference between the RBC cytoplasm and the surrounding medium [31], the Hb concentration can be calculated from the reconstructed RI distribution of RBCs. The cytoplasmic Hb content of individual RBCs was then obtained from the retrieved volume and Hb concentration (see Materials and Methods).

**Fig 3:**
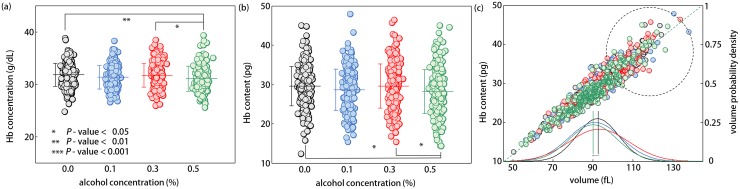
Biochemical parameters and the Hb content versus volume correlation map for RBCs of 0.0, 0.1, 0.3, and 0.5% ethanol concentrations (a) RBC Hb concentration, (b) RBC Hb content, and (c) correlation map between the Hb content and the volume, with the respective volume probability density curves. Each circle indicates an individual RBC measurement. The mean value of each morphological RBC parameter is denoted as a horizontal line with the vertical bar for the standard deviation.

Our results show that both mean concentration and content of Hb for RBCs exposed to a lethal concentration of ethanol (0.5% v/v) were significantly lower than those of RBCs in 0.0 and 0.3% ethanol groups [Fig 3a and 3b]. Meanwhile, these two biochemical parameters of RBCs in moderate ethanol groups (0.1 and 0.3%) were not statistically different from those of control RBCs. The mean Hb concentrations and Hb contents in RBC cytoplasm for the 0.0, 0.1, 0.3, and 0.5% ethanol groups were 31.8 ± 2.2, 31.3 ± 2.2, 31.7 ± 2.3, and 31.1 ± 2.4 g/dL and 29.6 ± 5.0, 28.6 ± 5.3, 29.6 ± 5.6, and 28.2 ± 5.6 pg, respectively. These observations strongly suggest the possibilities that ethanol-RBC interaction depletes the cytoplasmic Hb via certain ways, at least for situations where RBCs are exposed to ethanol concentration of more than 0.5%. The precedent paper based on Raman spectroscopy [19] reported the same tendency of Hb depletions in RBC cytoplasm under *in-vitro* ethanol treatments, but only in ranges far beyond the practically attainable blood alcohol concentrations (> 10% v/v).

In order to perform correlative analyses, the scatter plot of volume versus Hb content for every RBC is presented in Fig 3(c). Regardless of the ethanol concentration, all RBCs exhibited a linear positive correlation between the Hb content and the cell volume, indicating that the Hb concentration was not far altered upon ethanol exposure. Interestingly, there were only a few RBCs from the highest ethanol condition (0.5%, the green circles) inside the high Hb content vs volume range (the dashed black circle), compared to RBCs taken from the remaining ethanol treatment groups and the control group span over wide Hb content vs volume range.

### Modifications in Biomechanical Properties of RBC Membranes

**Fig 4:**
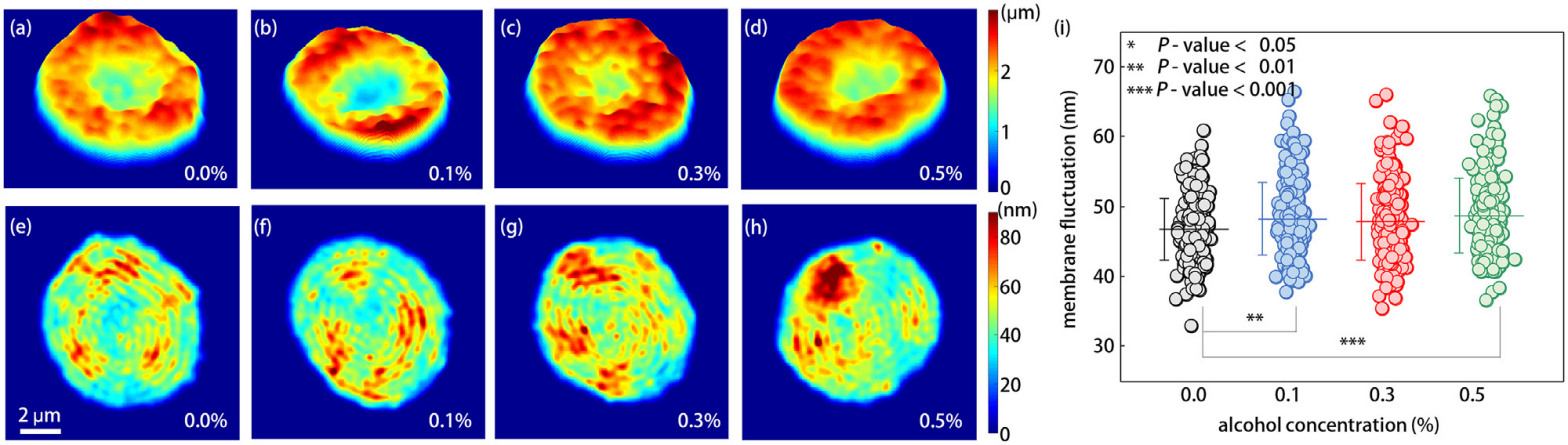
(a–d) 2-D membrane height maps of the representative individual RBCs in the (a) 0.0, (b) 0.1, (c) 0.3, and (d) 0.5% ethanol concentrations. (e–h) 2-D out-of-membrane fluctuation maps of the corresponding four RBCs in (a–d). (i) Dynamic membrane fluctuations for all measured RBCs. The colored circle indicates an individual cell measurement. The mean membrane fluctuation for each ethanol group is denoted as a horizontal line and standard deviation is represented by the vertical bar.

To investigate the effects of ethanol on RBC deformability, we measured dynamic membrane fluctuations of individual RBCs (see Materials and Methods). 2-D mean height maps and corresponding membrane fluctuation maps of four representative RBCs from the 0.0, 0.1, 0.3, and 0.5% ethanol groups are shown in Fig 4a–4d and 4e–4h, respectively.

**Fig 5:**
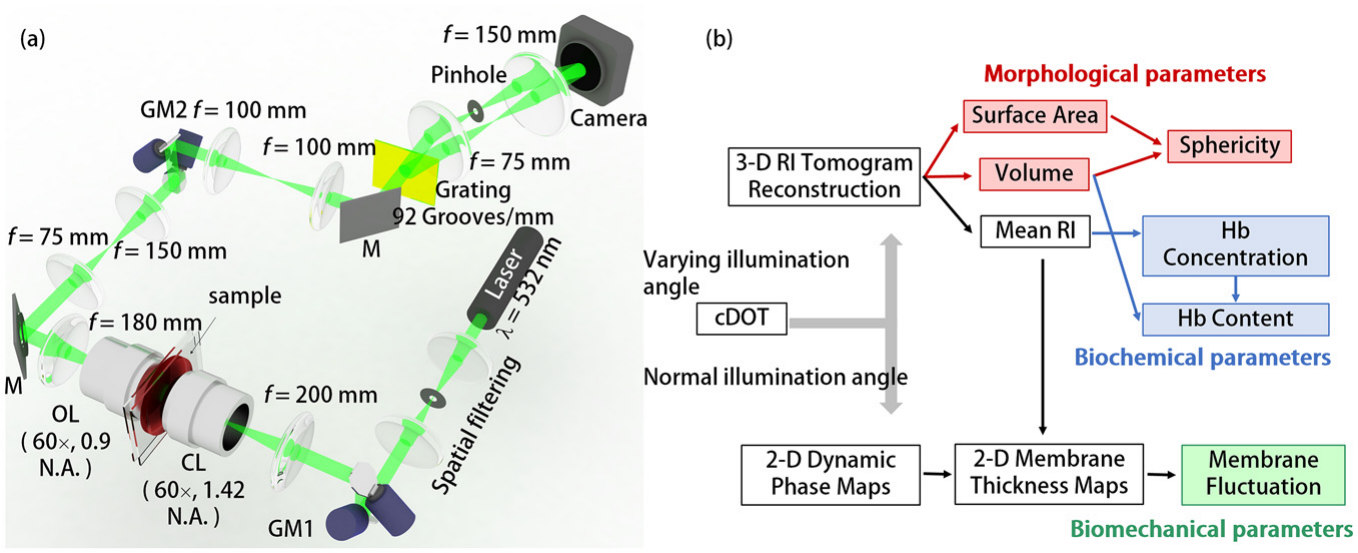
The cDOT setup and RBC analysis procedures (a) Optical setup of the cDOT. GM1-2: galvanometer mirrors, CL: condenser lens, OL: objective lens, M: mirror; (b) Schematic diagram of RBC analysis procedures for retrieving morphological (volume, surface area, and sphericity); biochemical (Hb concentration and Hb content); and biomechanical (membrane fluctuation) parameters.

In order to quantitatively address the alterations in RBC deformability due to ethanol exposure, membrane fluctuations averaged over cell area were obtained for individual RBCs, as shown in Fig 4(i). Amplitudes of membrane fluctuations of RBCs exposed to ethanol were significantly higher than those of control RBCs [Fig 5(i)]. The mean membrane fluctuations were 46.7 ± 4.4, 48.2 ± 5.2, 47.9 ± 5.5, and 48.7 ± 5.3 nm for RBCs in the 0.0, 0.1, 0.3, and 0.5% ethanol groups, respectively. Our results clearly indicate that ethanol enhanced the deformability of RBCs. The enhanced deformability of RBCs under *in-vitro* ethanol exposure was qualitatively consistent with other reports of elevated membrane fluidity of RBCs based on EPR spectroscopy [1, 5] and fluorescence anisotropy [7, 8] techniques; and of the increased elongation index in RBCs by ethanol (at least up to 4% v/v), using laser diffraction ektacytometry [6].

## Discussion

### Morphological modifications of RBCs by Ethanol

Our results showed that RBC morphology is altered by physiologically relevant ethanol concentrations (0.0–0.5%), which have been regarded too low to affect the RBC morphologies. First, the mean surface area of RBCs exposed to mild ethanol concentrations of 0.1 and 0.3% was statistically lower than that of control RBCs. RBCs in 0.3% ethanol group exhibited significantly elevated sphericities, compared to those of other three ethanol groups (*p* < 0.001). Whereas, both mean surface area and sphericity of RBCs in 0.5% ethanol group were not statistically different with those of control RBCs. Meanwhile, statistical analyses did not guarantee any difference among mean volumes of RBCs exposed to various ethanol concentrations.

It has been widely accepted that the elevated volume-to-surface area ratio of RBCs impairs a cell deformability [32, 33], implying the deteriorated deformability of RBCs under the influence of moderate ethanol (0.1 and 0.3% v/v) via losses of surface area. The minute changes in sphericity of RBCs in 0.5% ethanol group compared to control one are thought to be resulted from the exclusion of echinocytes in our RBC measurements (for details, see Materials and Methods), as predicted by a sudden appearance of echinocytes at this ethanol concentration. Interestingly, exactly this ethanol level, i.e. 0.5%, has been reported as a phase transition point where the filterability of RBCs through micro-size pores started to decrease [14].

Minute changes in RBC volume under *in-vitro* ethanol exposures up to 0.5% are also consistent with a previous report of undetectable MCV changes based on flow cytometer technique [15]. On the other hand, this result is in partial contrast to the widely known fact that RBCs in chronic alcoholics exhibit significantly large MCV [34]. However, the macrocytosis pertaining to this increased MCV in chronic alcoholics may not be involved in instantaneous effects of ethanol exposure on RBCs, such as was the case in our measurements, because macrocytosis in alcoholics is mainly associated with the folate deficiency induced by chronic alcohol ingestion [35, 36].

### Cytoplasmic alterations in RBCs by Ethanol: Hb Depletions in RBC Cytoplasm

Measurements of 3D RI tomograms of RBCs revealed that the mean Hb concentration of RBCs in lethal conditions (0.5% ethanol conc.) was significantly lower than that of control RBCs (adjusted *p* < 0.01). In general, decreases in Hb concentration of RBCs can be regarded as a consequence of a water influx or a loss of Hb in RBC cytoplasm. Of these possibilities, minute volume changes by ethanol [Fig 2(a)] suggest that the reduction in Hb concentration was due primarily to a loss of Hb molecules via certain processes, like Hb degradation or vesiculation. This assertion is also supported by substantial decreases in the Hb content of RBCs exposed to the highest ethanol concentration (0.5% v/v), even taking into account the hemolytic effects of ethanol. Ethanol-mediated Hb depletions in RBC cytoplasm have been reported using near-infrared Raman spectroscopy [19], but only under ethanol exposures of very high concentration which cannot be physiologically achieved (> 10% v/v). In comparison to such previous studies, however, observed Hb depletions at 0.5% ethanol concentration in this study may not only present plausible explanations about non-monotonic tendencies in both surface area and sphericity of RBCs, possibly via echinocytosis, but also present a clue which links the physiologically lethal condition to a specific ethanol concentration, i.e. 0.5%.

From a pathophysiological perspective, the loss of Hb molecules from the RBC cytoplasm associated with high ethanol concentrations, could counter-intuitively, be related to the increased prevalence of iron overload observed among heavy drinkers [37].

### Mechanical Modifications of RBC Membranes by Ethanol

The deformability of RBCs is crucial for their ability to pass through capillaries in microvasculature, and thus essential for oxygen transport to various organs. RBC deformability has been known to be determined by mechanical properties of the cell membrane cortex, viscosity of the cell cytoplasm, and its volume-to-surface area ratio [32, 33]. Mechanical properties (shear modulus, bending modulus and area expansion modulus) of the RBC cortex are governed by its spectrin network, composition of the lipid membrane, and the RBC shapes. Viscosity of the cell cytoplasm is primarily determined by the Hb concentration [38, 39]. Hence, deformability of RBCs, associated with the pathophysiology of various diseases, can be non-invasively and quantitatively accessed by probing dynamic membrane fluctuations, cytoplasmic Hb concentrations, and cell sphericities.

Our measurements show that ethanol treatments clearly enhanced membrane fluctuation in RBCs, as presented in Fig 4(i). This indicates that *in-vitro* ethanol treatments of RBCs indeed alter the mechanical properties of membrane in a way enhancing the cell deformability. This result is consistent with those of many previous studies using EPR resonance spectroscopy [1, 5, 6] or fluorescence anisotropy [7–11]. In particular, minute differences in fluctuation amplitudes among the 0.1, 0.3, and 0.5% ethanol exposure groups reflect the on/off effects of the ethanol on mechanical properties of the RBC membrane, at least in the range of blood alcohol concentration tested. In addition, lowered cytoplasmic viscosity caused by Hb depletions will also enhance a deformability of RBCs. On the contrary, elevated sphericity of RBCs via loss of surface area under mild ethanol exposures (0.1 and 0.3% v/v) may deteriorate the cell deformability at the same time. Although researchers generally agreed that the ethanol of mild concentration (0.0–0.5%) enhances the deformability of RBCs, the reduced deformability of RBCs at high ethanol concentrations (> 0.5%) has been reported in studies using a laser diffraction ektacytometer [6] and a RBC filteration method [14]. Possibly, the observed inconsistency of morphological (surface area and sphericiy), biochemical (Hb concentration and Hb content), and mechanical (membrane fluctuation) parameters affecting the RBC deformability upon ethanol exposures give rise to this reduced deformability of RBCs at high ethanol concentrations. Now, we even could present a plausible explanation of why the deteriorated deformability of RBCs exposed to high ethanol concentration has not been observed in studies using EPR spectroscopy or fluorescence anisotropy. That might be contributed by their inherent limitations in addressing morphological and biochemical alterations of RBCs; they measure the membrane fluidity. Accordingly, the morphological alteration of RBC by ethanol, like elevated volume-to-surface area ratio, irrelevant to membrane properties might be responsible for the reduced deformability of RBCs at ethanol concentration of more than 0.5%.

Interestingly, it has been reported that patients with chronic alcoholism exhibit stiffer RBC membranes than those of control RBCs, despite the instantaneous membrane fluidizing properties of ethanol molecules [3, 9, 13].

## Conclusions

In our study, individual RBCs subjected to *in-vitro* ethanol exposure were non-invasively and quantitatively measured using a QPI technique to investigate simultaneous effects of ethanol on morphological (volume, surface area, and sphericity); biochemical (Hb concentration and Hb content); and biomechanical (membrane fluctuation) properties, without complicated sample preparation. In particular, we primarily concerned on alterations in RBC characteristics by physiologically relevant concentration of ethanol from 0.0 to 0.5%, in which the effects of ethanol on RBC morphologies have not been systematically investigated. Simultaneously measured six parameters of individual RBCs also present a possible explanation mediating conflicts among the results of previous studies of exploiting different experimental techniques.

Our measurements showed that there were no abrupt and extreme translations in RBC morphologies, enough to be captured by naked-eye, by *in-vitro* ethanol treatments of up to 0.5% and these results are consistent with previously performed qualitative studies. With the help of individual cell measuring capability, however, cDOT technique allows us to conclude that the morphological, biochemical, and mechanical properties of RBCs are indeed affected by ethanol molecules in a dose-dependent manner. First of all, RBCs exposed to a lethal ethanol concentration of 0.5% exhibited statistically reduced cytoplasmic Hb concentration and Hb content, compared to those of control RBCs. These significant Hb depletions are thought to be related to sudden increases in both sphericity of RBCs and echinocyte population at this level of ethanol exposure. Meanwhile, deformability of RBCs started to be enhanced even at lower ethanol concentration, as reflected in elevated membrane fluctuation.

In view of the high sensitivity and the single-cell profiling capability rendered by the present QPI approach, it could potentially be used for the study of effects of other toxicities on blood cells, such as previous studies regarding red wine effects on hemorheological parameters [15, 40, 41]. In addition, recent advances in QPI methodologies including real-time measurements and visualization [42, 43], could also be further utilized to investigate the specific effect of ethanol on RBCs. As a practical issue, recently developed QPI unit [44, 45] and a simple illumination scheme using a DMD [46] could make this translate the present approach available to clinical laboratories by converting existing optical microscopes for QPI. Going forward, we envision that this QPI approach could have far-reaching applications in hematology, possibly in conjunction with newly emerging single-cell optical manipulation method [47].

## Materials and Methods

### Ethics statements and Sample preparations

Human blood studies were conducted according to the principles of the Declaration of Helsinki and were approved by the responsible ethics committee of Korea Advanced Institute of Science and Technology (KAIST) (IRB project number: KH2013-22, KH2015-37). The participant provided the written informed consent to participate to this study which was approved by the ethics committees and IRB.

All the RBCs measured in our experiments were collected from three healthy donors, according to the standard protocol [48]. In detail, bloods of 3 mL were collected from each healthy volunteer by venipuncture and readily transferred into EDTA anticoagulant tube at a KAIST clinic Pappalardo center. For optical measurement, collected blood (3 μL) was diluted with Dulbecco's Phosphate-Buffered Saline (DPBS) (Gibco^®^, New York, U.S.A.) without calcium and magnesium. Four ethanol concentrations were chosen considering relevant blood alcohol concentrations: 0.0% for normal (abstinence), 0.1 and 0.3% for binge drinking and severe alcohol intoxication, respectively, and 0.5% for an alcoholic or lethal condition. We exposed the diluted normal blood to the various alcohol concentrations for 10 min to provide adequate time for the ethanol molecules to interact with the lipid-bilayers in the RBC membranes. The treated blood was loaded between two coverslips (24 × 50 mm, C024501, Matsunami, LTD, Japan), and measurements were taken following a brief 20 min cell settling period. In each experimental trial, 40 RBCs of one blood donor were measured for each ethanol group. Then, for every blood donor, the same experiment was performed once more. In measuring cells, RBCs in discocyte shapes gently sedimented on a coverslip were selected; RBCs moving in translational motion, or RBCs undergoing echinocytosis, were excluded. After excluding erroneously measured RBCs, a total of 238, 236, 235, and 237 RBCs taken from the 0.0, 0.1, 0.3, and 0.5% ethanol concentration treatment groups, were analyzed. We observed that most of the cells used for the prepared ethanol concentrations were discocytes. It was reported that about 60% of discocytes were transformed into echinocytes when exposed to 2% v/v ethanol [49]. All measurements in each ethanol concentration were finished within 90 min. All experimental protocols were approved by the KAIST Institutional Review Board.

### Common-path diffraction optical tomography (cDOT)

To non-invasively and quantitatively measure 3-D RI tomograms and 2-D dynamic membrane fluctuations of individual RBCs simultaneously, we used cDOT [24, 25]. The experimental setup is shown in Fig 1(a). The cDOT employs the principle of common-path interferometry and optical diffraction tomography. A diode-pumped solid state laser (*λ* = 532 nm, 50 mW, Cobolt Co., Solna, Sweden) was used as an illumination light source. The beam size of a spatially filtered plane wave was further reduced using an aspheric tube lens (*f* = 200 mm) and a condenser objective lens (UPLFLN 60×, numerical aperture (N.A.) = 0.9, Olympus Inc., San Diego, CA, U.S.A.), before illuminating samples. The first two-axis galvanometer mirror (GVS012/M, Thorlabs, U.S.A.), GM1, controlled the illumination angle of the laser impinging on a sample. Samples loaded between two coverslips were positioned between the condenser lens and the imaging objective lens (PLAPON 60×, oil immersion, N.A. = 1.42, Olympus Inc., San Diego, CA, U.S.A.). The second galvanometer mirror, GM2, placed at the conjugated plane of the sample plane, compensated for changes in beam angle induced by GM1, such that the optic axis after GM2 remained fixed, regardless of the laser illumination angle at the sample plane.

Common-path interferometry was achieved employing diffraction phase microscopy [50, 51]. A diffraction grating (92 grooves/mm, #46–072, Edmund Optics Inc., NJ, U.S.A.) divided the laser beam into various diffraction orders, of which the 0^th^ order beam was spatially filtered using a pinhole (⊘25 μm, P25S, Thorlabs, U.S.A.), and served as the reference beam in the interferometry. The 1^st^ order beam, containing the optical phase information of a sample, served as a sample beam. These two beams interfered at the image plane and the resultant interferograms were recorded with a high-speed sCMOS camera (Neo sCMOS, ANDOR Inc., Northern Ireland, U.K.). All other diffracted beams were blocked. The total lateral magnification from the sample plane to the camera plane was ×250. The field of view was set at 14.3 × 13.31 μm^2^, corresponding to 528 × 512 pixels with a pixel size of 6.5 μm at the camera plane.

For reconstructing 3-D RI distributions of a sample, typically 300 interferograms were recorded at angles of illumination. From each interferogram, a light field consisting of both phase and amplitude images of a sample, was retrieved using a field retrieval algorithm [42, 52]. Employing the diffraction optical tomography algorithm [26, 53, 54], 3-D RI distribution of a sample was reconstructed from the complex light field images obtained with various angles of illuminations.

To measure 2-D dynamic membrane fluctuations of RBCs, the beam illuminating a sample was set to be parallel to the optic axis, and time-lapse phase images were measured [50, 51]. Normally, 300 consecutive interferograms were recorded with a frame rate of 125 Hz. From measured 2-D dynamic phase images Δ*φ*(*x*, *y*, *t*), RBC height image *h*(*x*, *y*) can be obtained using the following relation: *h*(*x*, *y*, *t*) = [*λ*/(2π·〈Δ*n*〉)]·Δ*φ*(*x*, *y*, *t*), where 〈Δ*n*〉 is the mean RI difference between the sample *n*(*x*,*y*,*z*) and the surrounding medium *n*_*medium*_: 〈Δ*n*〉 = 〈*n*(*x*,*y*,*z*)〉 − *n*_*medium*_, which was obtained from a 3-D RI tomographic measurement. Details about the field retrieval algorithms, the MatLab code for 3-D reconstruction, and the regularization algorithms can be found elsewhere [42, 55].

### Retrieval of RBC parameters

From the measured 3-D RI tomograms and 2-D dynamic phase maps, RBC parameters including morphological (cell volume, surface area, and sphericity); biochemical (Hb concentration and Hb content); and biomechanical (membrane fluctuation) parameters of individual RBCs were obtained, as summarized in Fig 1(b).

Cell volume *V* and surface area *S* were obtained from a measured 3D RI tomogram. Sphericity index *SI*, a measure of how closely a RBC resembles a sphere, is then calculated from a volume-to-surface area ratio [*SI* = π^1/3^(6*V*)^2/3^/*S*]. The *SI* has a value of ‘1’ for a perfect sphere and ‘0’ for a flat disk.

The Hb concentration of individual RBCs, [Hb], was calculated from a measured RI tomogram assuming that cytoplasm mainly consists of Hb solution. The mean RI difference between the cell and its surrounding medium 〈Δ*n*〉 is linearly proportional to [Hb] via the following equation: [Hb] = 〈Δ*n*〉/*α* where *α* is a refraction increment and has a value of 0.2 mL/g for Hb [56, 57]. Then, total Hb contents in a RBC was calculated by multiplying [Hb] by *V*.

Dynamic fluctuations in RBC membranes show strong correlation with cell deformability [58–60] and its alteration with pathophysiological conditions [48, 61–68]. To quantify cell deformability, root-mean-squared displacements of cell height fluctuations were calculated from measured dynamic 2-D phase images as follows, [〈(*h*(*x*, *y*, *t*) − 〈*h*(*x*, *y*, *t*)〉_temporal_)^2^〉_temporal_]^1/2^, from which RBC membrane fluctuation can then be obtained by averaging over the cell area. The details of the analysis procedure can also be found elsewhere [69].

### Statistical Analysis

One-way ANOVA was performed for comparing RBC parameters between various ethanol concentrations based on Bonferroni adjustments. All the presented *p*-values in texts were adjusted by Bonferroni correction and the numbers follow the ± sign are standard deviations. The threshold of family-wise error rate was set to be 0.05 in deciding statistical difference.
